# KRT84 is a potential tumor suppressor and good prognosis signature of oral squamous cell carcinoma

**DOI:** 10.1042/BSR20200187

**Published:** 2020-03-31

**Authors:** Yi Liu, Ronghua Li, Gang Ren

**Affiliations:** Department of Stomatology, Tianjin First Central Hospital, No. 24 Fukang Road, Nankai District, Tianjin 300192, P.R. China

**Keywords:** antigen processing and presentation signal pathway, KRT84, oral squamous cell carcinoma, prognosis

## Abstract

**Aims:** Oral squamous cell carcinoma (OSCC) is a common oral cancer; however, current therapeutic approaches still show limited efficacy. Our research aims to explore effective biomarkers related to OSCC.

**Main methods:** Gene expression profiles of paired OSCC tumor and paracancerous samples from The Cancer Genome Atlas (TCGA) were analyzed. mRNA and protein levels of KRT84 in OSCC cell line HSC-3 were measured by real-time quantitative polymerase chain reaction (RT-qPCR) and Western blot. KRT84 protein levels in OSCC tumor samples of different stages were determined by immunohistochemistry. Overall survival (OS) of OSCC samples was evaluated and association of multiple factors with OS was assessed.

**Key findings:** Compared with paracancerous samples, 4642 DEGs were identified in OSCC tumor samples. Among them, KRT84 expression level in OSCC tumor tissues was obviously decreased, which was validated in HSC-3 cells. KRT84 expression level showed decreasing tendency with the increase of tumor grade and stage. Patients with low KRT84 expression level had inferior OS independently of multiple factors. Besides, antigen processing and presentation pathway were significantly activated in OSCC samples with high KRT84 expression. Elevated KRT84 mRNA as well as protein levels were confirmed by RT-qPCR and Western blot in OSCC and normal cell lines, and immunohistochemistry in OSCC tumor and paracancerous tissues.

**Significance:** Our study suggests KRT84 as a tumor suppressor and good prognostic indicator for OSCC, which might be significant for OSCC diagnosis and treatment.

## Introduction

Oral squamous cell carcinoma (OSCC) is recognized as the most common phenotype of oral cancer, accounting for approximately 3% of the malignancies worldwide [1]. Annually, there are more than 300,000 patients diagnosed with OSCC worldwide, which is within the top 10 morbidity of all cancers and continues to increase [2–4]. Consumptions of tobacco and alcohol are responsible for almost 90% of the OSCC cases, while oral hygiene, human papillomavirus, and dental status are also considered as the risk factors [5]. Currently, the treatment modalities of OSCC mainly include surgery, radiotherapy, chemotherapy, or combinations of these approaches [6]. However, despite of the substantial advances in therapeutic approaches of OSCC and improvement in survival status, the overall 5-year survival is only 50–60%, which remains unchanged in recent years. The relatively low overall 5-year survival is partially caused by the limited efficacy of current therapeutic approaches in OSCC, especially for the metastatic OSCC, which is believed to be one of the major risk factors for OSCC mortality [6–8]. Considering the low survival rate and unoptimistic status of OSCC treatment, it is imperative to explore more effective biomarkers for further understanding of OSCC pathogenesis and facilitation of its therapeutic strategies.

It is known that genetic alteration is the critical characterization of OSCC, involving in multistep processes [9]. Among the genetic factors, the differential expression of genes is widely studied. For example, Chang et al. showed that the genes NPM, CDK1, and NDRG1 were up-regulated in OSCC samples, while CHES1 expression level was down-regulated [10]. The aberrant expression of PA28γ gene was suggested to be a prognostic biomarker of OSCC patients [11]. Peroxiredoxin-2 (PRDX-2) and zinc-alpha-2-glycoprotein (ZAG) were proved to be up-regulated in OSCC patients, which might be adopted as potential biomarkers for OSCC detection [12]. On the other hand, epigenetic factors also play a pivotal role in OSCC, such as DNA methylation. DNA methylation was previously reported to down-regulate the promoter activity of human cathelicidin antimicrobial peptide (CAMP) gene, which was associated with the carcinogenesis of OSCC [13]. Besides, the down-regulations of LATS1/2 genes caused by promoter methylation were also observed in OSCC patients [14]. Although numerous genetic biomarkers were identified, the knowledge on the detailed biological function and in-depth analysis are still limited for many of them [15], and some of the researches mixed the primary sites of OSCC. Therefore, identification of other biomarkers associated with site-specific OSCC closely and investigation on the inherent biological mechanism warrant further research.

In the present study, we compared the gene expression levels between OSCC and paracancerous samples from TCGA, and identified KRT84 as a potential tumor suppressor. Survival analysis was performed to study the association of KRT84 expression with the prognosis of OSCC patients from TCGA, which was further validated in the Gene Expression Omnibus (GEO) dataset. The present study should be helpful for OSCC early diagnosis and treatment.

## Materials and methods

### Data source

The gene expression profiles of OSCC were obtained from The Cancer Genome Atlas (TCGA, https://www.cancergenome.nih.gov) and Gene Expression Omnibus (GEO, https://www.ncbi.nlm.nih.gov/geo/). The TCGA dataset included 145 OSCC samples with the primary site arising on the tongue, and 14 of them had paired paracancerous samples. The GEO dataset (Access number: GSE41613) includes 97 OSCC samples.

### Differential expression analysis

We used the DESeq2 package in R software to screen the differentially expressed genes (DEGs) in the 14 OSCC tumor samples compared with paracancerous samples. The adjusted *P* value < 0.05 and |log2 Fold Change| > 2 were selected as the thresholds for significantly differential expression.

### Cell culture

The human OSCC cell line HSC-3 and normal human oral gingival epithelial cell line HOEC were obtained from the cell bank at Chinese Academy of Sciences (Shanghai, China). The HSC-3 cells were cultured in K-SFM medium (Gibco, U.S.A.) containing 10% fetal bovine serum (FBS, Hyclone, U.S.A.), and the HOEC cells were cultured in DMEM medium (Gibco, U.S.A.) containing 10% FBS (Hyclone, U.S.A.). All the cells were cultured at 37°C in humidified air with 5% CO_2_ and passaged after reaching confluence with 0.25% trypsin-EDTA solution (Sigma-Aldrich, St. Louis, U.S.A.).

### Real-time quantitative polymerase chain reaction (RT-qPCR)

The total RNA of HSC-3 and HOEC cells was extracted using TRIzol reagent (Invitrogen, California, U.S.A.). The cDNA was synthesized with Moloney murine leukemia virus reverse transcriptase (Roche, Basel, Switzerland) with random primers and subjected to PCR products using rTaq polymerase (Takara Bio Inc, Shiga, Japan). RT-qPCR was performed using SYBR Green PCR mix (Invitrogen, California, U.S.A.) according to the manufacturer’s instructions. The primer pairs used for amplification were shown as follows: KRT84 F: 5′-CCAACGTGGATACCCTAACT-3′; R: 5′-CTCTGAGATGTGCGACTGC-3′; GAPDH F: GGTGAAGGTCGGTGTGAACG; R: CTCGCTCCTGGAAGATGGTG. The expression of KRT84 was normalized to the endogenous reference gene GAPDH, and the relative expression was analyzed using the 2^−ΔΔCt^ method.

### Western blot

The HSC-3 and HOEC cells were lysed in RIPA buffer, and total protein was quantified using bovine serum albumin (BSA, Boster, Wuhan, China). After being added with 5× loading buffer and boiled for 5 min, the proteins were electrophoresed on 10% sodium dodecyl sulfate-polyacrylamide gel electrophoresis (SDS-PAGE) and then transferred onto polyvinylidene fluoride (PVDF) membranes (Millipore, U.S.A.). The membranes were blocked with 1× Blotto and incubated with primary antibodies (anti-KRT84 antibody, 1:1000, Abcam, U.S.A.; anti-GAPDH antibody, 1:1000, Abcam, U.S.A.) at 4°C overnight. After incubation with horseradish peroxidase (HRP)-labeled goat anti-rabbit IgG (abcam, U.S.A.) at room temperature for 1.5 h, the membranes were developed with Western Lightning™ Chemiluminescence Reagent (PerkinElmer, U.S.A.). The intensity of protein was measured by using LabWorks™ (UVP, U.S.A.), with GAPDH as the internal control.

### Immunohistochemistry

The OSCC tumor samples including stage I, II, III and IV were fixed in 4% paraformaldehyde, and transparentized by xylene. After paraffin embedding, the samples were sliced into sections of 5 μm. Then dewaxing and antigen retrieval were performed. After blocking with normal goat serum and incubation at room temperature for 20 min, the primary antibody was added, followed by an incubation at 4°C overnight. Then the secondary antibody was added for an incubation at 37°C for 20 min. The sections were developed in DAB, counterstained with hematoxylin, and sealed with resinene. A total of 5 fields were randomly selected, photographed under the optical microscope (BX51T-PHD-J11, Olympus, Tokyo, Japan), and analyzed by Image-Pro Plus (MediaCybernetics, Silver Spring, MD, U.S.A.). Written informed consent was obtained from all participants and our study was approved by the Ethics Committee of Tianjin First Central Hospital.

### Functional enrichment analysis

The functional enrichment analysis was performed by using Gene Set Enrichment Analysis (GSEA) software, and the significantly enriched pathways were screened with *P* < 0.05 as threshold.

### Statistical analysis

Two-sided *t* test was performed to determine the significance of difference in gene expression level between two groups. The overall survival (OS) of OSCC samples was evaluated by Kaplan–Meier method, and log-rank test was used to determine the significance of difference in OS between two groups. Association of multiple factors with the OS of OSCC samples was assessed based on the multivariate Cox regression analysis. The significance of difference in mRNA and protein expression levels between the OSCC cells and normal human oral gingival epithelial cells was determined by *t*-test. Comparisons of mean intensity in immunohistochemistry among samples of different stages were performed by one-way analysis of variance (ANOVA). All statistical analyses were conducted in R software (version 3.2.2), with *P* < 0.05 as the threshold for statistical significance.

## Results

### KRT84 was a potential tumor suppressor in OSCC

**Figure 1:**
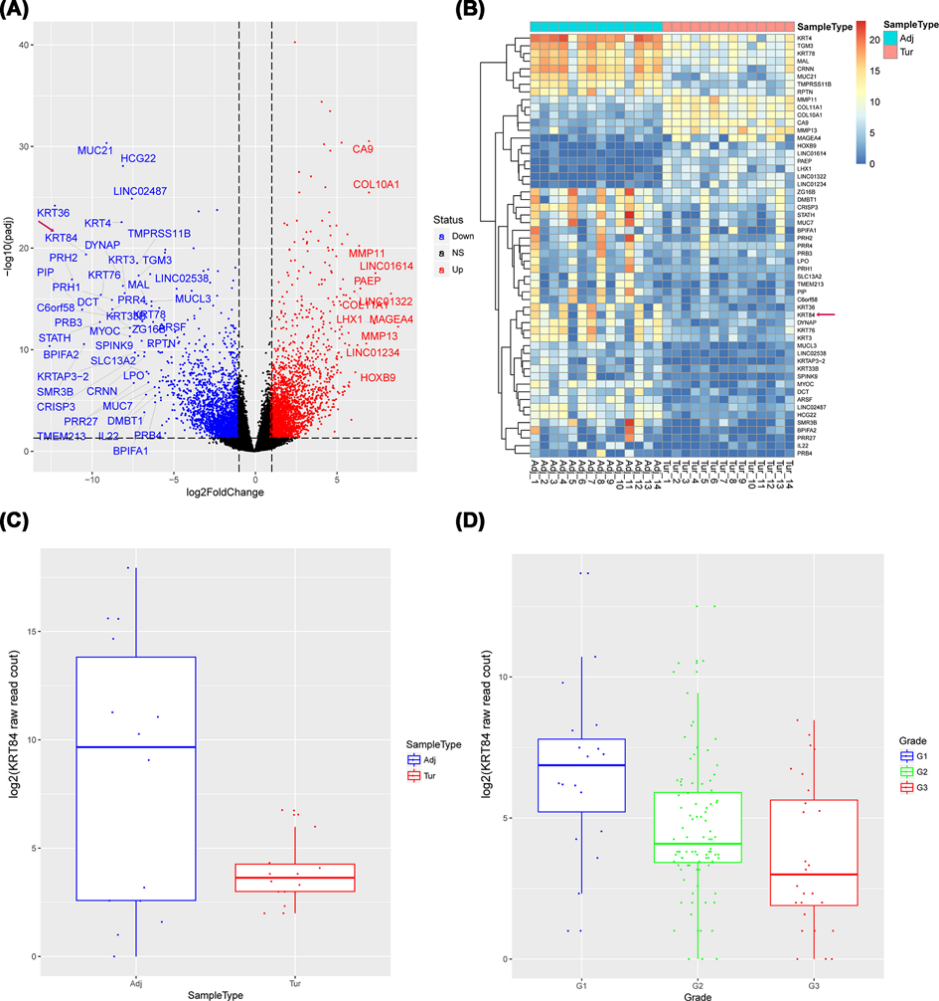
KRT84 might be a potential tumor suppressor in OSCC (**A**) The volcano plot of the differentially expressed genes (DEGs) between the 14 paired OSCC tumor and paracancerous samples. (**B**) The heatmap of 55 DEGs with |log2 Fold Change| > 2 in 14 paired OSCC tumor and paracancerous samples. (**C**) The expression level of KRT84 in OSCC tumor samples was significantly lower than that in the paracancerous samples (*P* = 4.23 × 10^−20^). (**D**) With the increase of OSCC tumor grade, the KRT84 expression level was reduced (G1 vs. G2, 6.68 vs. 4.60, *P* = 7.89 × 10^−3^; G1 vs. G3, 6.68 vs. 3.59, *P* = 6.79 × 10^−4^; G2 vs. G3, 4.60 vs. 3.59, *P* = 0.041).

After analyzing the gene expression profiles of OSCC samples, a total of 4642 DEGs were identified between in OSCC tumor samples compared with paracancerous samples, among which 2254 were down-regulated and 2388 were up-regulated in OSCC tumor samples (Figure 1A). The 55 DEGs with |log2 Fold Change| > 2 were marked in the volcano plot, and the relevant heatmap of gene expression in 14 paired cancer and paracancerous samples was shown in Figure 1B. The results showed that the expression level of KRT84 in OSCC tumor samples was significantly lower than that in paracancerous samples. Thus, we selected gene KRT84 for further analysis. As shown in Figure 1C, the KRT84 expression level in OSCC tumor samples was strikingly decreased compared with the paracancerous samples (*P* = 4.23 × 10^−20^). Moreover, as shown in Figure 1D, the KRT84 expression level was obviously reduced with the increase of OSCC tumor grade (G1 vs. G2, 6.68 vs. 4.60, *P* = 7.89 × 10^−3^; G1 vs. G3, 6.68 vs. 3.59, *P* = 6.79 × 10^−4^; G2 vs. G3, 4.60 vs. 3.59, *P* = 0.041). These results indicated that KRT84 was a potential tumor suppressor in OSCC.

### Validation of decreased KRT84 expression in OSCC

**Figure 2:**
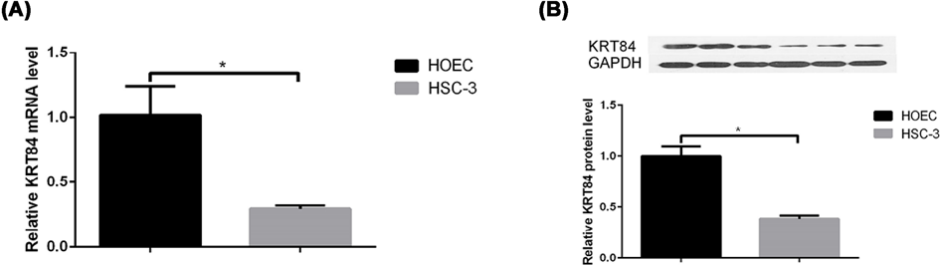
KRT84 mRNA and protein expression levels in human OSCC cell line HSC-3 was significantly lower than that in normal human oral gingival epithelial cell line HOEC (**A**) Histogram of KRT84 mRNA expression levels between human OSCC cell line HSC-3 and normal human oral gingival epithelial cell line HOEC. *, *P* < 0.05 vs. the control group. (**B**) Histogram of KRT84 protein expression levels between human OSCC cell line HSC-3 and normal human oral gingival epithelial cell line HOEC. *, *P* <0.05 vs*.* the control group.

**Figure 3:**
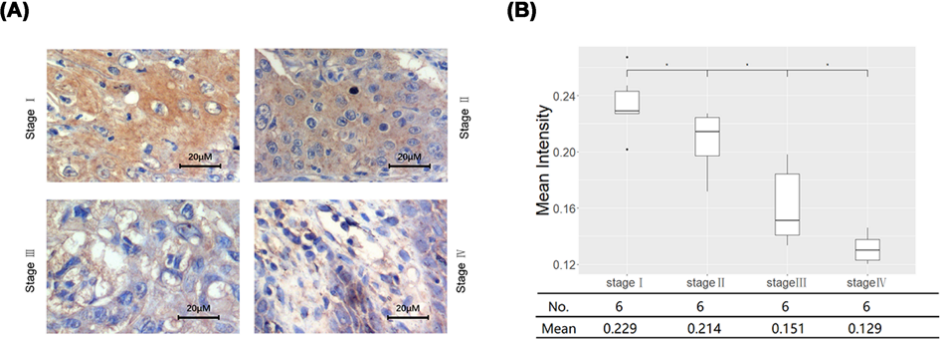
The protein expression level of KRT84 in OSCC tumor tissues was significantly decreased with the increase of tumor stage (**A**) Immunohistochemical staining of OSCC tissues from stage I to stage IV. (**B**) Mean intensity of OSCC tissues from stage I to stage IV.

The mRNA and protein expression levels of KRT84 in human OSCC cell line HSC-3 and normal human oral gingival epithelial cell line HOEC were determined by RT-qPCR and Western blot. As shown in Figure 2A, the KRT84 mRNA expression level in HSC-3 cells was significantly lower than that in HOEC cells (*P* < 0.05). Compared with the HOEC cells, the protein expression level of KRT84 in HSC-3 cells was also manifestly decreased (*P* < 0.05, Figure 2B). Immunohistochemistry was performed to measure the KRT84 protein levels in OSCC tumor samples from stage I to IV. It was found that the mean intensity from stage I to IV showed an obviously decreasing tendency (Figure 3). These results further validated the down-regulation of KRT84 in OSCC cells, as well as the decreasing tendency of KRT84 expression in OSCC tissues with the increase of tumor stage.

### Down-regulated KRT84 was associated with inferior prognosis of OSCC patients

**Figure 4:**
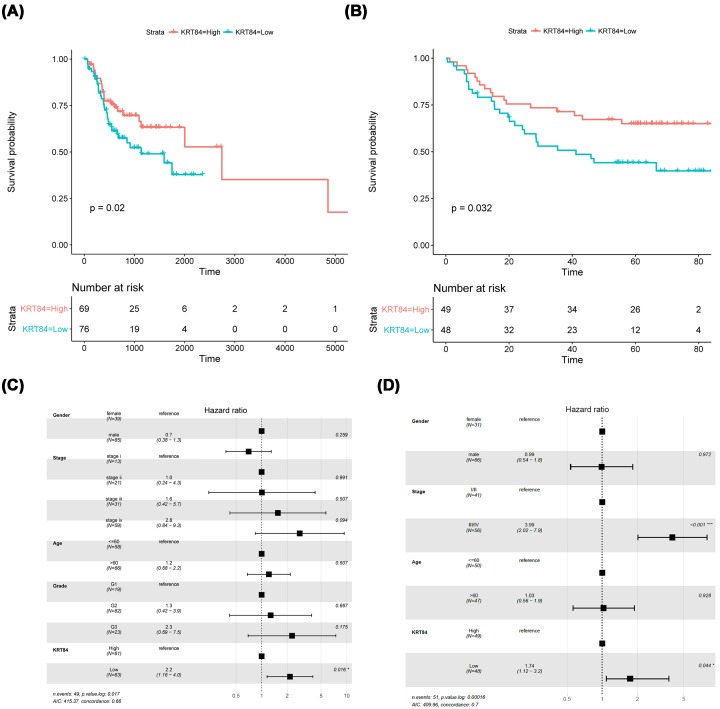
KRT84 expression level could influence the OSCC prognosis independently (**A**) In TCGA cohort, the patients with high KRT84 expression level had longer overall survival (OS) compared with the patients with low KRT84 expression level (*P* = 0.02). (**B**) In GEO cohort, the patients with high KRT84 expression level presented longer OS than the patients with low KRT84 expression level (*P* = 0.032). (**C**) KRT84 could influence the OSCC prognosis independently in TCGA cohort (HR = 2.2, *P* = 0.016). (**D**) KRT84 could also influence the OSCC prognosis independently in GEO cohort (HR = 1.74, *P* = 0.044).

According to the median of KRT84 expression level, the OSCC samples from TCGA database were divided into KRT84 high and KRT84 low groups. The survival analysis showed that the patients with high KRT84 expression level presented longer OS in comparison with the patients with low KRT84 expression level (*P* = 0.02, Figure 4A). This association of KRT84 expression with OSCC prognosis was further validated in GEO dataset, which showed a consistent result (*P* = 0.032, Figure 4B). Then multivariate Cox regression analysis was performed on the TCGA dataset with multiple factors as variables, including gender, age, tumor stage, tumor grade, and KRT84 expression level. It was found that KRT84 could affect the OSCC prognosis independently (HR = 2.2, *P* = 0.016, Figure 4C). Multivariate Cox regression analysis was also conducted on the GEO dataset with factors including gender, age, tumor stage, and KRT84 expression level as variables, and the result confirmed that KRT84 was an independent prognostic factor in OSCC (HR = 1.74, *P* = 0.044, Figure 4D).

### KRT84 might involve in OSCC carcinogenesis and prognosis through mediating antigen processing and presentation pathway

**Figure 5:**
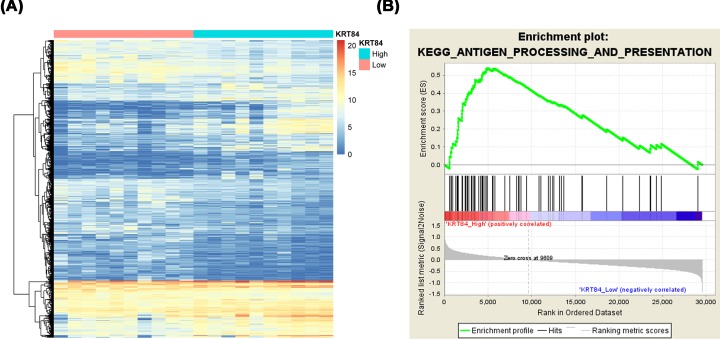
KRT84 might involve in the carcinogenesis and prognosis of OSCC through reulating antigen processing and presentation pathway (**A**) The expression heatmap of 2301 DEGs in KRT84_High and KRT84_Low groups. (**B**) The antigen processing and presentation pathway was significantly activated in KRT84_High samples.

The top 10 samples with the highest and lowest expression levels of KRT84 from TCGA were selected, and named as KRT84_High and KRT84_Low groups, respectively. After analyzing the gene expressions between KRT84_High and KRT84_Low groups, we identified 2301 DEGs between the two groups (Figure 5A). GSEA showed that the antigen processing and presentation pathway was significantly activated in KRT84_High samples (Figure 5B), indicating that KRT84 might involve in the carcinogenesis and prognosis of OSCC by regulating the antigen processing and presentation pathway. There are another three pathways including terpenoid backbone biosynthesis, linoleic acid metabolism, and arachidonic acid metabolism that significantly activated in KRT84_High OSCC sample group, and their detailed information were provided in Supplementary Table S1.

## Discussion

In the present study, we comprehensively investigated the biomarkers associated with the occurrence, development and prognosis of OSCC through bioinformatic analysis and experimental work. The differential expression analysis of 14 paired OSCC tumor and paracancerous samples showed that compared with the paracancerous samples, 2254 genes were down-regulated and 2388 genes were up-regulated in OSCC tumor samples. Among these DEGs, the expression level of KRT84 in the tumor tissues of OSCC was obviously lower than that in the paracancerous tissues. Experimental work including RT-qPCR and Western blot further confirmed the down-regulated mRNA and protein levels of KRT84 in OSCC cells in comparison with normal human oral gingival epithelial cells. Moreover, the KRT84 expression level presented an obviously decreasing tendency with the increase in tumor grade. Immunohistochemistry showed that with the increase in OSCC tumor stage, KRT84 protein level was also decreased.

Keratin, as the important constitution of skin, nail, and hair, is a protein of the intermediate filament family and consists of type I and type II subunits [16,17]. KRT84, also known as type II hair keratin Hb4, is able to form nail and hair with type I keratins [18]. It is known that keratin protects the epithelial cells against mechanical stress, reflects diverse epithelial functions and participates in multiple biological processes including translation, malignant transformation, and proliferation [19]. An accumulating evidence has indicated the association of keratin with tumor. For example, keratin was proved to be the common marker for identification of disseminated tumor cells (DTC) and circulating tumor cells (CTC) [20]. It was reported that keratin expression was decreased in the metastatic progression of breast cancer [20]. Besides, Paccione et al. found the expression level of differentiation-specific keratins was reduced if the tumor cells experienced epithelial-to-mesenchymal transition (EMT) [21]. But to our knowledge, the expression level of KRT84 in OSCC has not been studied yet. Our research first reported the down-regulation of KRT84 in OSCC.

Survival and Cox regression analyses of the OSCC samples from TCGA revealed that patients with low KRT84 expression level had inferior OS, which was independent of multiple factors including gender, age, tumor stage, and tumor grade. Further analysis on the validation set from GEO showed consistent result. The prognostic values of keratins in cancers have been widely studied. Keratin 17 expression occurred in approximately half of the gastric cancer patients in a previous study, which was positively correlated with tumor progression and poor prognosis [22]. In hepatocellular carcinoma, keratin 19 was associated with larger tumor size as well as more invasive features, indicating worse prognosis [23]. Soeth et al. found that keratin 20 positivity in the blood and bone marrow predicted inferior prognosis in pancreatic adenocarcinomas [24]. In spite of the extensive studies on the prognostic value of keratins, the effect of down-regulated KRT84 as poor prognostic indicator in OSCC has rarely been investigated. Our study provided reference for the prognostic role of KRT84 in OSCC.

Functional enrichment analysis demonstrated that in the OSCC samples with high KRT84 expression, the antigen processing and presentation signal pathway was significantly activated. In the epithelial cells from tumor tissues, the co-expression of keratin and major histocompatibility complex (MHC) class I antigens, which played an essential role in antigen processing and presentation, was observed [25,26]. It is known that antigen processing and presentation enhancement facilitates immune recognition of tumors, which inhibits the tumor growth [27]. Based on these researches, we inferred that elevated KRT84 expression level might be accompanied by strong expression of MHC class I molecules, which inhibited the tumor development and contributed to superior prognosis by enhancing the antigen processing and presentation process. Linoleic acid metabolism and arachidonic acid metabolism are two acid metabolism-related pathways that were also significantly activated in KRT_High OSCC tumor groups. Acid metabolism have been extensively proved to be associated with cancer initiation and progression [28–30] by previously studies. Linoleic acid and arachidonic acid were the two acids that specifically identified in the present study, whose metabolism might be dysregulated by aberrant KRT84 expression, so this should also be potential pathways by which KRT84 could regulate OSCC progression.

## Conclusion

In conclusion, our study showed that KRT84 expression level was down-regulated in OSCC, which presented a decreasing tendency with the increase of tumor grade, and was associated with poor prognosis. In addition, KRT84 might involve in the pathogenesis and development of OSCC via interacting with the antigen processing and presentation signal pathway. Our research provided a novel potential biomarker for OSCC, which should be helpful for OSCC early diagnosis and treatment.

## Supplementary Material

Supplementary Table S1

## Abbreviations

CTC: circulating tumor cells
DTC: disseminated tumor cells
EMT: epithelial-to-mesenchymal transition
MHC: major histocompatibility complex
OS: overall survival
OSCC: oral squamous cell carcinoma
RT-qPCR: real-time quantitative polymerase chain reaction
TCGA: The Cancer Genome Atlas
