# Supplementary material for: KRT84 is a potential tumor suppressor and good prognosis signature of oral squamous cell carcinoma
Source: Biosci Rep. 2020 Mar 31;40(4):BSR20200187. doi: 10.1042/BSR20200187 (PMC7109001; doi:10.1042/BSR20200187)
Supplement: Supplementary Table S1 [file BSR-2020-0187_supp.pdf]

Table S1 Significantly activated pathways in KRT84_High OSCC tumor samples.

Pathway Name	SIZE	NES	NOM p-val
KEGG_ANTIGEN_PROCESSING_AND_PRESENTATION	80	1.725685	0.01568
KEGG_TERPENOID_BACKBONE_BIOSYNTHESIS	15	1.656415	0.01711
KEGG_LINOLEIC_ACID_METABOLISM	29	1.518516	0.025896
KEGG_ARACHIDONIC_ACID_METABOLISM	58	1.519905	0.035857
